# *Streptomyces* sp. AC04842: Genomic Insights and Functional Expression of Its Latex Clearing Protein Genes (*lcp1* and *lcp2*) When Cultivated With Natural and Vulcanized Rubber as the Sole Carbon Source

**DOI:** 10.3389/fmicb.2022.854427

**Published:** 2022-05-02

**Authors:** Ann Anni Basik, Chanaporn Trakunjae, Tiong Chia Yeo, Kumar Sudesh

**Affiliations:** ^1^Ecobiomaterial Research Laboratory, School of Biological Sciences, Universiti Sains Malaysia, Gelugor, Malaysia; ^2^Sarawak Biodiversity Centre, Kuching, Malaysia; ^3^Kasetsart Agricultural and Agro-Industrial Product Improvement Institute (KAPI), Kasetsart University, Bangkok, Thailand

**Keywords:** degradation, gene expression, homolog, latex clearing protein, latex rubber, vulcanized rubber

## Abstract

Rubber-degrading Actinobacteria have been discovered and investigated since 1985. Only recently, through the advancement of genomic sequencing and molecular techniques, genes and pathways involved in rubber degradation are being revealed; however, the complete degradation pathway remains unknown. *Streptomyces* sp. AC04842 (JCM 34241) was discovered by screening at a Culture Collection Centre in Sarawak for *Actinomycetes* forming a clear zone on natural rubber latex agar. *Streptomyces* is a dominant and well-studied soil bacterium playing an important role in soil ecology including carbon recycling and biodegradation. *Streptomyces* sp. AC04842 draft genome revealed the presence of 2 putative latex clearing protein (*lcp*) genes on its chromosome and is closely related to *Streptomyces cellulosae*. Under the *Streptomyces* genus, there are a total of 64 putative *lcp* genes deposited in the GenBank and UniProt database. Only 1 *lcp* gene from *Streptomyces* sp. K30 has been characterized. Unlike *Streptomyces* sp. K30 which contained 1 *lcp* gene on its chromosome, *Streptomyces* sp. AC04842 contained 2 *lcp* genes on its chromosome. *Streptomyces* sp. AC04842 lcp1 and lcp2 amino acid sequences showed 46.13 and 69.11%, respectively, similarity to lcp sequences of *Streptomyces* sp. K30. Most rubber degrading strains were known to harbor only 1 *lcp* gene, and only recently, 2–3 *lcp* homologs have been reported. Several studies have shown that *lcp*-homolog expression increased in the presence of rubber. To study the expression of *lcp1* and *lcp2* genes for *Streptomyces* sp. AC04842, the strain was incubated in different types of rubber as the sole carbon source. In general, the *lcp1* gene was highly expressed, while the *lcp2* gene expression was upregulated in the presence of vulcanized rubber. Mixtures of natural and vulcanized rubber did not further increase the expression of both *lcp* genes compared with the presence of a specific rubber type. In this study, we paved the way to the exploration of lcp homologs and their function in degrading different types of rubber.

## Introduction

Raising awareness related to rubber wastes problems has not been as successful as plastic wastes; however, both issues are just as critical. In addition to the principle rubber wastes known to date, i.e., tires, the growing wastes generated from rubber gloves, especially in this pandemic era, are unavoidable. Knowledge on the fate of rubber materials in nature is still limited. The rate of decomposition ranges from a year (latex glove) to up to 2,000 years (tires) to decompose (Basik et al., 2021b). More important than ever, we must increase our knowledge and continue to discover, learn, and explore strains suitable for treating rubber wastes in a safe way. In this study, we focused on biodegradation, the use of microorganisms capable of utilizing rubber as the sole carbon and energy source. Rubber-degrading strains produce enzymes known as latex clearing protein (Lcp), mainly present in Actinobacteria and rubber oxygenases (RoxA and RoxB) found in Gram-negative rubber degraders (Basik et al., 2021a,b). These microorganisms not only improve the rate of rubber degradation but also efficiently utilize these complex polymers and release safe by-products back into the environment as mineral salts, biomass, water, and carbon dioxides.

Rubber-degrading strain *Streptomyces* sp. AC04842 (JCM 34241) was discovered by screening an Actinobacteria Culture Collection from Sarawak Biodiversity Centre (SBC) (Basik et al., 2021a). *Streptomyces* sp. AC04842 was isolated from a soil sample collected from the Serapi Mountain in Kubah National Park, Kuching, Sarawak, East Malaysia, in the year 2007. As a dominant soil bacterium, *Streptomyces* are well studied due to their easy cultivation, suitability as a heterologous protein host, and wide metabolic ability, including carbon recycling. *Streptomyces* also play key roles in soil ecology because of their ability to scavenge nutrients and, in particular, to hydrolyze a wide range of polysaccharides (cellulose, chitin, xylan, and agar) and other natural macromolecules (Barka et al., 2016).

*Streptomyces* sp. AC04842 is a mesophilic strain, growing well by day 3 and at up to 55°C. Taxonomic studies suggest that this strain is a distinct species, closely related to a *Streptomyces cellulosae* JCM 4462 (reported in 1914), capable of producing fungichromin (Harrison et al., 1986). Putative genes involved in rubber degradation were also found in *Streptomyces* sp. AC04842 genome.

Several quantitative *lcp* gene expression studies have been carried out using quantitative polymerase chain reaction (qPCR) studies. *Nocardia* sp. strain NVL3 *lcp* gene showed 1,596-fold higher in the presence of synthetic rubber (Linh et al., 2017). *Actinoplanes* strain OR16 showed an increase of 22.2-, 17.1-, and 335-fold for *lcp1*, *lcp2*, and *lcp3* genes, respectively, when cultivated in NR (Gibu et al., 2020), while *Streptomyces* sp. K30 and *Gordonia polyisoprenivorans* VH2 showed increased *lcp* gene expression when cultivated with poly(*cis*-1,4-isoprene) compared with those grown with glucose or sodium acetate (Bröker et al., 2008; Yikmis et al., 2008).

*Streptomyces* sp. AC04842 genome contained 2 *lcp* genes on its chromosome. The *lcp* genes were located apart on different contigs. Following our previous research question “whether the incorporation of Lcp homologs impacts the ability of the strain to utilize and degrade different rubber products?” we decided to explore the functional expression of 2 *lcp* homologs for *Streptomyces* sp. AC04842 (Basik et al., 2021a). In *Streptomyces* sp. AC04842, putative Lcp (*lcp1* and *lcp*2) gene expression increased when cultivated with rubber (natural and vulcanized) as the sole carbon and energy source, with *lcp1* gene being highly expressed compared with *lcp2* gene under all conditions.

## Materials and Methods

*Streptomyces* sp. AC04842 taxonomic position was determined based on morphological characteristics and genome-based analysis. Putative rubber degradation genes in *Streptomyces* sp. AC04842 were also identified. To study the ability of this strain in utilizing different rubber materials as the sole carbon source, fresh latex, latex glove, and tire samples were used. Fresh latexes were harvested from the rubber tree *Hevea brasiliensis.* Latex gloves manufactured using natural rubber were purchased. Two different sizes of tire samples were used. For *lcp* gene expression studies, tire powder (400–600 μm), instead of tire granules, was used to mimic the microparticle pollution in the environment (refer to the ‘‘RNA Extraction and Quantitative PCR’’ section). Larger tire sample sizes, i.e., tire granules (1–3 mm), were used for rubber utilization studies (refer to the ‘‘Utilization of Rubber Materials’’ section). Smaller tire samples with larger surface areas were favorable for biodegradation studies. Being difficult to cut and dissolve, sources of tire samples from tire recycling factories would ease research related to tire biodegradation. There, tires are separated into tire granules, steel-free tire granules, and shredded tire pieces (accessed on 15 October 2021).^1^ Microplastics from tires are also the second-largest polluters (28%) in the ocean (Basik et al., 2021b). The largest polluter is synthetic textiles (35%).

### Strain Identification

*Streptomyces* sp. AC04842 morphology was observed on yeast malt extract (ISP2) agar, and their spore-forming structures were observed on soil extract agar (SEA) (Hamaki et al., 2005). Their spore-forming structures were observed directly using a 50 × long-distance lens (Olympus LMPLFLN; Olympus, Tokyo, Japan). Molecular identification was made based on the amplification of the 16S rDNA gene using primer 27F (5′-AGAGTTTGATCMTGGCTCAG-3′) and 1492R (5′-TACGGYTACCTTGTTACGACTT-3′) with the following parameters: 5 min at 96°C, 30 cycles of 45 s at 96°C, 2 min at 55°C, 4 min at 72°C, and the final extension for 7 min at 72°C.

Amplified products were sent to Apical Scientific Sdn. Bhd., Selangor, Malaysia, for sequencing. Sequence quality was checked using Sequence Scanner version 2.0 (Applied Biosystems, Waltham, MA, United States) (ThermoFisher Scientific, 2021) and assembled by manual alignment (cap contig) using BioEdit software version 7.0.5.3 (North Carolina State University, Raleigh, NC, United States) (Hall, 1999). The sequence’s blast homology was compared with the UniProt database (Bateman et al., 2021). Nucleotide sequences were translated into amino acids using ExPASy (Gasteiger et al., 2003) to identify the open reading frame (ORF). ORF was subjected to BlastP (GenBank, Maryland, United States) to obtain Protein Blast homology.

### Genomic DNA Isolation, Nucleotide Sequencing, and Sequence Analysis

*Streptomyces* sp. AC04842 was cultivated in 20 ml ISP2 broth (Shirling and Gottlieb, 1966) in a 250 ml flask at 28°C and 180 rpm for 3 days. Genomic DNA (gDNA) was extracted and purified using a method from Moore et al. (2008). The DNA quantity and quality were verified by spectrophotometric means (Eppendorf Biospectrophotometer basic). The purified DNA samples were subsequently sent for genomic sequencing using Illumina MiSeq by service provider BioEasy Sdn. Bhd. SnapGene Viewer version 5.1.3.1^2^ was used to identify *lcp* genes and other related rubber degrading genes including oxidoreductase α-subunit (OxiA) and oxidoreductase β-subunit (OxiB).

#### Genome Attributes and Annotation

The draft genome for *Streptomyces* sp. AC04842 was annotated using RAST server (Aziz et al., 2008), PATRIC server (Davis et al., 2020), and NCBI Prokaryotic Genome Annotation Pipeline (PGAP) (Li et al., 2020). Gene function annotation for COG was carried out using egg-NOG mapper v.2 (Cantalapiedra et al., 2021). Clusters of orthologous groups (COGs) were generated by comparing the predicted and known proteins in all completely sequenced microbial genomes to infer sets of orthologs. Each COG consists of a group of proteins found to be orthologous across at least three lineages and likely corresponds to an ancient, conserved domain (Tatusov et al., 2000). To determine related genes/protein and location in a pathway, the KEGG Pathway database was used (Kanehisa et al., 2015). Clustered regularly interspaced short palindromic repeats (CRISPRs) determined from PATRIC server were used to depict the genome readability for bacteriophage exposure and genome editing (Davis et al., 2020). To predict the antibiotic-resistant genes, antibiotic-resistant homologs search was carried out in the CARD database (Alcock et al., 2020). Drug targets were identified using PARTIC server (Davis et al., 2020) and verified using DrugBank 5.0 (Wishart et al., 2018).

The strains’ ability to produce secondary metabolites was predicted using The Antibiotics and Secondary Metabolite Analysis Shell (antiSMASH) 6.0 software (Blin et al., 2021).

To determine the location of protein-coding genes (CDS) and ORF on the draft genome, sequence contigs were viewed using The SEED Viewer (Overbeek et al., 2013) and SnapGene Viewer (see text footnote 2, accessed on 5 April 2020).

Protein-coding genes and amino acid sequence for Lcp, 1-oxidoreductase beta subunit (OxiB), and 1-oxidoreductase alpha subunit (OxiA) were verified through the presence of ribosome-binding site (RBS) and comparison with sequences in the publicly available databases UniProt (UniProt Consortium, 2021) and GenBank. The prediction of twin-arginine translocation (Tat) signal peptides was carried out using TatP 1.0 (Bendtsen et al., 2005). ExPASy (SIB Swiss Institute of Bioinformatics, Lausanne, Switzerland) was used to determine the predicted protein molecular weight (*M*_*w*_) and their theoretical isoelectric point (pI) (Gasteiger et al., 2003).

### RNA Extraction and Quantitative PCR

Spore suspension (1 × 10^6^ spores/ml) for S*treptomyces* sp. AC04842 cells was inoculated into 50 ml ISP2 media at 28°C and 180 rpm for 3 days. Then, 1 ml of cell pellets was transferred into 50 ml MSM supplemented with either 0.2% (w/v) glucose, 0.5% (w/v) fresh latex, or 0.2% (w/v) tire powder at 28°C and 80 rpm (Coenen et al., 2019).

Aliquots of 1 ml were harvested after 3 (inoculated with glucose) or 8 (inoculated with rubber sample) days. Cell pellets were resuspended in 100 μl TE buffer (10 mM Tris, 1 mM EDTA, pH 8.0) with 10 mg/ml lysozyme and incubated overnight at 37°C (Thronton and Basu, 2011). Total RNA was then isolated using the Nucleozol and purified using RNAeasy mini kit (Qiagen, Germany) according to the manufacturer’s protocol. RNA was transcribed into complementary DNA (cDNA) with 200 U M-MuLV reverse transcriptase. Additionally, a control approach without reverse transcriptase was prepared to exclude gDNA contaminations. The primers were designed using Primer3Plus software^3^ (Coenen et al., 2019). Lcp1 gene primers Slcp1f (5′-CCAAGAGCGTCTACTGGTC-3′) and Slcp1r (5′-GAGTTCGGAAAGGTCGTAG-3′), Lcp2 gene primers Slcp2f (5′-GTTTCAGCGTACCAAGTGAT-3′) and Slcp2r (5′-GTAATCTCCGCCTGTTGAT-3′), and 16S rDNA gene primers S16f (5′-CGGATACTGATCGCCTTGGG-3′) and S16r (5′-CACACTGGGACTGAGACACG-3′) were used.

Amplification efficiency of the designed primer was determined by generating a standard curve. Standard curve is made using 5 concentration points by serially diluting known sample concentration (10-fold). The PCR efficiency is calculated using the following formula:


PCRefficiency=10-(-1/slope)1×100

Values of PCR efficiency ranging between 90 and 110% are preferred, as efficiency value 100% indicates that the number of newly formed DNA amplicons is doubled in each cycle.

To determine the specificity of the designed primers, melt curves were analyzed. The presence of a single peak shows that the primer specifically binds to the target sequence. In addition, gel analysis showing the presence of a single band represents the amplification of one PCR product at the expected range.

Quantitative polymerase chain reaction (qPCR) was performed in a total reaction volume of 50 μl containing 1 μl of synthesized cDNA, 0.5 nM of primers, and 25 μl of the SYBR Green PCR Master Mix (Applied Biosystems, Inc., United States) using the QuantStudio™ 5 Real-Time PCR System (Thermo Fisher Scientific, Waltham, MA, United States). The reaction conditions of qPCR were as follows: hold stage at 50°C for 2 min and 95°C for 2 min, 40 cycles of 95°C for 15 s and 60°C for 1 min. For negative control, no cDNA was added, and therefore, no amplification should be observed during qPCR.

The relative quantification method was used to analyze the data of qPCR. It is expressed as the fold change between the study samples: (i) rubber degrading strains incubated with rubber materials as the sole carbon source and control and (ii) rubber degrading strains incubated with glucose as the sole carbon source. To normalize the target gene expression, 16S cDNA was used as the reference gene. The cycle threshold (Ct) was obtained, and the relative comparison of each target gene was analyzed using the following formula (Livak and Schmittgen, 2001):


Relativefoldchange=2Ct-ΔΔ

where

Ct = number of cycles required for the fluorescent signal to cross the threshold

ΔCt = Ct target gene – Ct reference gene

ΔΔCt = ΔCt sample – ΔCt control

All data were expressed as mean ± standard deviation.

### Utilization of Rubber Materials

To study the ability of *Streptomyces* sp. AC04842 in utilizing different rubber materials as the sole carbon and energy source, fresh latex, rubber gloves, and tire samples were used. Fresh latex was harvested from 5-year-old rubber trees at a rubber plantation site at Kulim, Kedah. The latex was brought back and left to solidify at room temperature. Solid latex pieces were then cut into ∼1.0 cm × 1.0 cm pieces. Steel-free tire granules (1.0–3.0 mm) were obtained from a tire recycling factory in Kedah (Gcycle Tyre Recycling). Latex gloves (PRO-CARE), disposable and non-powdered, were used in these studies. Latex gloves were cut into strips of ∼0.5 cm × 1.0 cm.

For short-term evaluation under laboratory conditions, antimicrobial substances from the latex glove and tire granules were removed prior to cultivation. The rubber materials were treated with chloroform as follows: 1 g of sample with 100 ml for 12 h. During this period, the solvent was replaced 1–2 times with fresh chloroform. The treated material was left to dry, then sterilized by autoclaving, and subsequently used as a carbon source (Berekaa et al., 2000). No changes on the surface (cracks and holes) of the rubber material were observed using scanning electron microscope (SEM) before and after chloroform treatment.

Pre-culture of actively growing strains was cultivated in ISP2 broth. The culture (1 ml) was then transferred into 250 ml test flasks containing 50 ml MSM and 0.5% (w/v) rubber material (Berekaa et al., 2000). The inoculated flasks were then incubated at 28°C and 180 rpm for 30 days. Test flasks without culture were used as control. All sample studies were carried out in triplicates.

#### Morphological Observation Using Scanning Electron Microscope

After 60 days, inoculated rubber samples were harvested and air-dried for direct observation. To observe the surface of the tested rubber material, the strain biofilm and mycelia on the rubber particles were removed by rinsing the rubber materials with distilled water and then immersing them in 96% ethanol for 1 h before air-drying them at ambient temperature. Both washed and unwashed samples were prepared using the hexamethyldisilazane (HDMS) method prior to SEM viewing. Samples were placed into shell vials (1.8 ml) and fixed by immersing them in *McDowell-Trump* fixative solution, prepared in 0.1 M phosphate buffer (pH 7.2) at 4°C for at least 2 h. The samples were then washed with 0.1 M phosphate buffer (pH 7.2) for 10 min. This step was repeated two times. Samples were then post-fixed by immersing them in 1% (v/v) osmium tetroxide prepared in 0.1 M phosphate buffer (pH 7.2) for 1 h. This step was carried out in the chemical hood. Then, the samples were washed by immersing them in distilled water for 10 min (2 times). Dehydration of the samples was carried out using ethanol. First, the samples were immersed in 50% ethanol for 15 min, followed by 75% for 15 min, 95% for 15 min (2 times), and finally 100% for 20 min (3 times). The dehydrated samples were then immersed in 1 ml HDMS for 10 min. HDMS acts as a drying agent. HDMS solutions were discarded, and the samples were left to dry in the desiccator. Dried samples were then mounted onto an SEM stub using double tape, coated with gold using Quorum Q150T S sputter coater (Quorum Technologies Ltd., East Sussex, United Kingdom) for observation using SEM Quanta FEG 650 (Thermo Fisher Scientific, Waltham, MA, United States).

#### Attenuated Total Reflection-Fourier Transform Infrared

Test strain biofilm and mycelia were removed from the rubber samples. This was done by rinsing the rubber materials with distilled water and then immersing them in 96% ethanol for 1 h before air-drying them at ambient temperature. Attenuated total reflection-Fourier transform infrared (ATR-FTIR) is used to determine the formation of new, or disappearance of, functional groups in the polymer units of the samples by observing the presence, increase, and decrease in C=C, C–C, and C–H bonds (Basik et al., 2021a). Rubber samples (e.g., 5 mg of fresh latex pieces, 1 mg of latex glove strips, and 2 mg of tire granules) inoculated and non-inoculated were analyzed using ATR-FTIR Spectrum 400 (Perkin Elmer), equipped with ATR ranging from 4,000 to 650/cm (4/cm resolution).

## Results

Under this section, *Streptomyces* sp. AC04842 morphology and genome were successfully characterized. Rubber degrading ability for this strain was also evaluated, together with the functional expression of its putative *lcp1* and *lcp2* genes under different culture conditions.

### *Streptomyces* sp. AC04842 Strain Characterization

**FIGURE 1:**
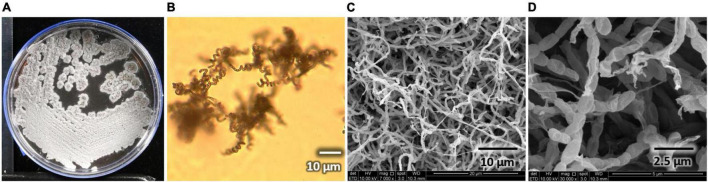
*Streptomyces* sp. AC04842 morphology. **(A)** Surface colony morphology on yeast malt extract (ISP2) agar; **(B)** spore-forming structures viewed using a light microscope at 750× magnification on soil extract agar (SEA); **(C)** abundant and long spore chains were observed using a SEM (SEM Quanta FEG 650); **(D)** chains of smooth, oblong spores were seen using a SEM (SEM Quanta FEG 650).

*Streptomyces* sp. AC04842 has a cream-colored surface and reverse color on yeast malt extract (ISP2) agar. Once matured, its surface area is grayish with abundant sporulation, and brownish pigmentation is produced on ISP2 agar. Melanin polymers possess diverse molecular structures (typically black or brown) formed by oxidative polymerization of phenolic and indolic compounds. They are not essential for the organisms, but they play a crucial role in improving their survival and competitiveness (Barka et al., 2016). *Streptomyces* sp. AC04842 colonies are wrinkled with regular shape. It has an external long spiral spore chain with oblong spores (∼1 μm). *Streptomyces* sp. AC04842 is a mesophilic strain that can be cultivated up to 55°C on ISP2 agar. *Streptomyces* sp. AC04842 grows actively by day 3 and starts to plateau on day 5 onward when cultivated in ISP2 broth at 28°C and 180 rpm. Strain morphology can be seen in Figure 1.

### *Streptomyces* sp. AC04842 Taxonomic Position

**FIGURE 2:**
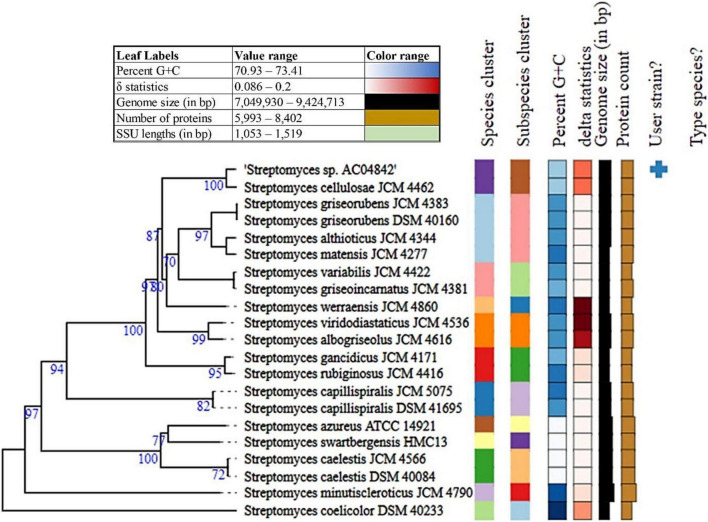
Phylogenetic analysis of *Streptomyces* sp. AC04842 draft genome with other closely related *Streptomyces* strains genomes using TYGS analysis. Tree inferred with FastME 2.1.6.1 (Lefort et al., 2015) from GBDP distances calculated from genome sequences. The branch lengths are scaled in terms of GBDP distance formula d5. The numbers above branches are GBDP pseudo-bootstrap support values > 60.00% from 100 replications, with an average branch support of 83.30%. The tree was rooted at the midpoint (Farris, 1972).

To further establish the strain position at the species and subspecies level, genome analysis for *Streptomyces* sp. AC04842 was conducted using average nucleotide analysis (ANI), digital DNA-DNA hybridization (dDDH), and genome-based phylogeny (Figure 2). According to the current bacterial taxonomy, the projected and generally accepted dDDH and ANI values are 70 and 95–96%, respectively, between genomes of the same species (Chun et al., 2018).

The mean nucleotide identity of orthologous gene pairs shared between *Streptomyces* sp. AC04842 and *S. cellulosae* strain NBRC 13,027 genome was compared using FastANI (KBase Software). An ANI estimate of 99.13% suggests that *Streptomyces* sp. AC04842 belongs to the same species as *S. cellulosae*.

Digital DNA-DNA hybridization values were computed using Type Strain Genome Server (TYGS) using formula d4, which is independent of the genome length (>20% of genome length) and is thus robust against the use of incomplete draft genomes (Auch et al., 2010). *Streptomyces* sp. AC04842 dDDH was 93.10% to *S. cellulosae* JCM 4462. G+C content for *Streptomyces* sp. AC04842 was less than 1.00% with other *Streptomyces* strains that are within species variation.

Taking together the (i) 16S rRNA gene-based method, (ii) average nucleotide identity (ANI), (iii) *in silico* DNA-DNA hybridization (DDH) estimate method, and (iv) genome relatedness and phylogenetic analysis of *Streptomyces* sp. AC04842, data support that it belongs to the same species as *S. cellulosae*.

### *Streptomyces* sp. AC04842 Genome Annotation

The draft genome for *Streptomyces* sp. AC04842 (JADBHJ000000000) has a size of almost 8 kb (7,831,043 bp) with a DNA G+C content of 71.8 mol%. The draft genome has a capacity for 6,998 protein-encoding genes and 15 rRNAs [5S (8), 16S (4), 23S (3)] and 80 tRNAs. For *Streptomyces* sp. AC04842, the subsystem coverage is 28%, which contributes to a total of 438 subsystems out of 6,998 CDS predicted by RAST server (Supplementary Figure 1).

Predicted proteins were further analyzed into different functional classes based on the Cluster of Orthologous Groups (COGs). A total of 5,790 proteins were mapped to unique COGs that were further classified into metabolism (35.06%), cellular processes and signaling (18.32%), information storage and processing (20.36%), and poorly characterized (18.53%) functional classes. The remaining 7.72% proteins were assigned to more than one COGs categories and were grouped into multiple COG (Supplementary Figure 2).

### *Streptomyces* sp. AC04842 Specialty Genes

Three CRISPR arrays were detected as sites accessible for bacteriophage exposure and genome modification.

Six antibiotic-resistant genes were predicted using CARD database (strict criteria), with functions in antibiotic resistance toward aminoglycoside (ARO:3003395), eflamycin (ARO:3003359 and ARO:3003368), macrolide (ARO:3003748 and ARO:3000463), and aminocaumarin (ARO:3002522).

Ten drug targets were verified using DRUGBANK, namely, one putative gene encoding Recombinase A, RecA (Pfam: PF00154), 1 putative gene encoding 4-hydroxymandelate synthase (Glyoxalase, Pfam: PF00903), 1 putative gene encoding 3-dehydroquinate dehydratase (DHquinase_II, Pfam: PF01220), 1 putative gene encoding cell division protein FtsZ [Tubulin (Pfam: PF00091) and FtsZ_C (Pfam: PF12327)], 1 putative gene encoding elongation factor tau [GTP_EFTU_D2 (Pfam: PF03144) and GTP_EFTU_D3 (Pfam: PF03143)], 1 putative gene encoding xylose isomerase [AP_endonuc_2 (Pfam:PF01261)], 1 putative gene encoding endo-1,4-beta-xylanase a [Glyco_hydro_10 (Pfam: PF00331) and Ricin_B_lectin (Pfam: PF00652)], 1 putative gene encoding propionyl-CoA carboxylase complex B subunit [Pfam: Carboxyl_trans (PF01039)], 1 putative gene encoding beta-glucosidase A, and 1 putative gene encoding uncharacterized protein [SnoaL_3 (Pfam: PF13474)].

*Streptomyces* sp. AC04842 draft genome contained 30 genes related to the chloroaromatic degradation pathway genes (*catA*, *catF*, *catI*, *catJ*, and *PCAH*) and p-hydroxybenzoate degradation genes (*pobA*, *HT*) (Göbel et al., 2002). Some soil microbes or plant pathogens are known to have this ability, as plants contain significant levels of natural phenolic compounds essential for reproduction and growth and a defense mechanism against pathogens (Felestrino et al., 2020).

Specialty genes for *Streptomyces* sp. AC04842 including CRISPR sites, antibiotic-resistant genes, drug target genes, and chloroaromatic degradation are listed in Supplementary Table 1.

### *Streptomyces* sp. AC04842 Secondary Metabolite Potential

A total of 16 secondary metabolite biosynthetic gene clusters (BGCs) were predicted using AntiSMASH software (Table 1). No putative macrolide compound was detected, suggesting that *Streptomyces* sp. AC04842 does not produce fungichromin as reported for *S. cellulosae*. Two putative carotenoid compounds (36 and 27% similar to other carotenoid compounds) were detected. Carotenoids protect cells against photooxidative damage and hence found important applications in the environment, food and nutrition, disease control, and as potent antimicrobial agents (Kirti et al., 2014). Putative BGCs encoding novel lanthipeptide class I were detected. Polycyclic lantibiotics are possible solutions to antibiotic resistance as they are protease-resistance, highly stable, and target-specific (Alkhalili and Canbäck, 2018).

**TABLE 1:** *Streptomyces* sp. AC04842 secondary metabolite biosynthetic gene clusters (BGCs) were predicted using antiSMASH 6.0 server (antiSMASH bacterial version with strict detection).

Region	Type	From (bp)	To (bp)	Most similar known cluster		Similarity
Contig 5	Type 2 polyketide synthase (T2PKS)	1	57,566	Spore pigment	Polyketide	83%
Contig 8	Terpene	19,643	40,728	Albaflavenone	Terpene	100%
Contig 9	Terpene	35,798	56,799	Cyslabdan	Terpene	81%
Contig 5	Ectoine	34,631	45,029	Ectoine	Other	100%
Contig 32	T2PKS, oligosaccharide	1	48,271	Grincamycin	Polyketide:Type II + Saccharide:Hybrid/tailoring	82%
Contig 34	RiPP-like	19,112	29,327	Informatipeptin	RiPP:Lanthipeptide	57%
Contig 42	Lanthipeptide-class-i	10,020	34,542			
Contig 57	Siderophore	26,706	38,478	Desferrioxamine B/Desferrioxamine E	Other	83%
Contig 77	Terpene	19,676	33,199	Carotenoid	Terpene	36%
Contig 117	Phenazine	1	19,807	Endophenazine A/endophenazine B	Other:Phenazine	44%
Contig 125	Siderophore	12,018	21,493			
Contig 140	Redox-cofactor	3,829	17,961	Lankacidin C	Non-ribosomal Peptide (NRP) + Polyketide	26%
Contig 188	Terpene	1	13,456	Hopene	Terpene	53%
Contig 234	Terpene	1	9,983	Carotenoid	Terpene	27%
Contig 256	Terpene	1	8,011	Geosmin	Terpene	100%
Contig 263	RiPP-like	1,504	7,766			

*RiPP, Ribosomally synthesized and posttranslationally modified peptides (RiPP)-like.*

### *Streptomyces* sp. AC04842 Latex Clearing Protein Operon

**FIGURE 3:**
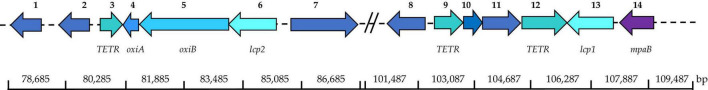
Location of putative *lcp1* and *lcp2* and adjacent putative genes in *Streptomyces* sp. AC04842 located on the chromosome based on the MeDuSa assembly. Scaffold 48: 1, LD-carboxypeptidases (EC 3.4. 17.13); 2, lipase 2 (EC 3.1.1.3); 3, transcriptional regulator, TETR-family; 4, isoquinoline 1-oxidoreductase alpha subunit (*oxiA*); 5, isoquinoline 1-oxidoreductase alpha subunit (*oxiB*); 6, latex clearing protein gene 1 (*lcp1*); 7, long-chain fatty acid CoA ligase (EC 6.2.1.3). Scaffold 5: 8, alpha-methylacyl-CoA racemase (EC 5.1.99.4); 9, transcriptional regulator, TetR-family; 10, FIG01128310: hypothetical protein; 11, alcohol dehydrogenase (EC 1.1.1.1); 12, transcriptional regulator, TETR-family; 13, latex clearing protein gene 2 (*lcp2*); 14, oxygenase MpaB family protein. Putative gene location was detected using the SEED Viewer (Overbeek et al., 2013) and SnapGene Viewer. The putative gene identity was compared with UniProt (UniProt Consortium, 2021) and GenBank databases.

Latex clearing protein operon has been reported for other *Streptomyces* rubber degraders including *Streptomyces* sp. K30 and *Streptomyces* strain CFMR 7 (Bröker et al., 2008; Nanthini et al., 2017). *Streptomyces* sp. K30 contained 1 *lcp* gene, while *Streptomyce*s strain CFMR 7 had 3 *lcp* homologs located on the chromosome. In this study, we described the presence and position of 2 *lcp* homologs for *Streptomyces* sp. AC04842 (Figure 3).

NCBI Conserved Domain Database (CDD) search shows that *Streptomyces* sp. AC04842 *lcp* homologs (*lcp1* and *lcp2* genes) belong to the DUF2236 domain-containing protein (accession pfam 09995) similar to *Streptomyces* sp. K30 Lcp, which is involved in the cleavage of poly(*cis*-1,4-isoprene), yielding isoprenoid aldehydes and ketones. DUF2236 is an uncharacterized protein conserved in bacteria found in various hypothetical bacterial proteins and has no known function. This family contains a highly conserved arginine and histidine that may be active site residues for a yet unknown catalytic activity.

*Streptomyces* sp. AC04842 is the first rubber-degrading strain known to have 2 *lcp* gene homologs that are located far apart and are not detected on the same contig. Putative *lcp1* gene was located on contig 20 (59,155 bp), while putative *lcp2* gene was located on contig 42 (46,197 bp) (Supplementary Figure 4). To predict the location of *lcp1* gene and *lcp2* gene in the chromosome, *de novo* genome assembly was conducted using *Streptomyces* sp. SID 4956 complete genome (WWIT01000818) *via* MeDuSa server (Figure 3). No complete genome was available for *S. cellulosae*. Blast homology of *Streptomyces* sp. AC04842 16S rRNA gene and *Streptomyces* sp. SID 4956 whole-genome sequence was 99.20%. Based on the *de novo* assembly of *Streptomyces* sp. AC04842 and *Streptomyces* sp. SID 4956, the longest contig was 7,525,530 bp (draft genome total length was 7,831,450).

The *lcp* gene nucleotide sequences were analyzed using UniProt Blast. The *lcp1* gene (1,233 bp) showed 93.66% blast homology to Lcp of *Streptomyces* sp. 4F (ALV53416). The *lcp2* gene (1,224 bp) showed 69.14% blast homology to Lcp of *Streptomyces* sp. LCIC4 (BAK52801). Both Lcp homolog sequences contained the 13-residue-long highly conserved region. Amino acid sequences of lcp1 and lcp2 showed 77% similarity.

Putative *lcp1* gene (MT664881) of *Streptomyces* sp. AC04842 is located on contig 20 (48,655–49,887 bp), and the CDS translation results in 411 amino acids, encoding a protein with a theoretical mass of 44.4 kDa and 6.15 pI. Tat signal peptide cleavage site was predicted between 48 and 49 bp (AGA-AA). Putative ribosomal site (RBS) was detected at 11 bp upstream from the putative start codon. The lcp1 amino acid sequence showed 46.13% similarity to the Lcp of *Streptomyces* sp. K30 (AAR25849).

Putative *lcp2* gene (MT664882) of *Streptomyces* sp. AC04842 is located on contig 42 (28,891–30,114 bp), and the CDS translation results in 408 amino acids, encoding a protein with a theoretical mass of 44.4 kDa and 5.38 pI. Tat signal peptide cleavage site was predicted between 30 and 31 bp (ARA-RS). Putative ribosomal site (RBS) was detected at 7 bp upstream from the putative start codon. The lcp2 amino acid sequence showed 69.11% similarity to the Lcp of *Streptomyces* sp. K30 (AAR25849.1).

Putative genes encoding oxidoreductase α-subunit (OxiA) and oxidoreductase β-subunit (OxiB) proteins were first discovered in 2005, downstream of *lcp* gene in *Streptomyces* sp. strain K30. Oxidoreductase complex (OxiAB) contributed to the size of the clear zone formation and facilitated the strain to oxidize, resulting in aldehydes from polyisoprene-degraded products (Rose et al., 2005).

Oxidoreductase β-subunit (OxiB) belongs to the molybdopterin-binding domain of aldehyde dehydrogenase (pfam02738) under Ald_Xan_dh_C2 superfamily (accession cl29417).

Putative *oxiB* gene (MZ711223) was detected 6 bp downstream of *lcp2* gene on contig 42 (7,368,051–7,370,360 bp) with the length of 2,310 bp encoding a putative protein of 770 amino acids with a theoretical mass of 81.9 kDa and a pI 6.54. OxiB amino acid sequence of *Streptomyces* sp. AC04842 blast homology was 99.37% identity to OxiB of *S. cellulosae* (GHE38178).

Oxidoreductase α-subunit (OxiA) belongs to (2Fe-2S)-binding protein (domain architecture ID 11449880) under CoxS superfamily (accession COG2080). (2Fe-2S)-binding protein is the small subunit of a dehydrogenase or oxidoreductase enzyme complex such as carbon monoxide dehydrogenase and isoquinoline 1-oxidoreductase. It contains a 2Fe-2S ferredoxin-type domain, which binds 2Fe-2S clusters.

Putative *oxiA* gene (MZ615456) was detected downstream of *oxiB* gene (20 bp) and *lcp2* gene on contig 42 (7,367,558–7,368,031 bp) with the length of 474 bp encoding a putative protein of 70 amino acids with a theoretical mass of 7.7 kDa and a pI of 4.99. OxiA amino acid sequence of *Streptomyces* sp. AC04842 blast homology was 99.48% identity to oxiA of *S. cellulosae* (GHE38187).

### Putative Genes Involved in *Streptomyces* sp. AC04842 Rubber Degradation

Putative genes involved in rubber degradation were identified based on studies made on several rubber-degrading strains; *Streptomyces coelicolor* 1A, *Nocardia* sp. 835A, *Steroidobacter cummioxidans* sp. nov., strain 35Y, *G. polyisoprenivorans* VH2, and *Nocardia nova* SH22a (Tsuchii et al., 1985; Tsuchii and Takeda, 1990; Bode et al., 2000; Rose and Steinbüchel, 2005; Hiessl et al., 2014; Luo et al., 2014; Sharma et al., 2018). Based on this, putative genes that may participate in the rubber degradation in *Streptomyces* sp. AC04842 were identified. TetR-family is a regulatory mechanism that induces the production of Lcp protein during the metabolization of rubber (Yikmis et al., 2008; Coenen et al., 2019; Oetermann et al., 2019). A total of 77 putative TetR-family genes were detected. Three genes located nearby *lcp* genes may be related to the rubber degradation in *Streptomyces* sp. AC04842 (Figure 3).

Four putative gene coding Tat proteins were identified. Lcp secreted outside the bacterial cell through the Tat pathway breaks down the polyisoprene polymer into isoprenoid acids, which are then converted into acyl-CoA thioester by an acyl-CoA synthase (1 candidate gene). Acyl-CoA thioesters are further catabolized by an acyl-CoA dehydrogenase (5 candidate genes). The 2,4-dienoyl-CoA reductase (4 candidate genes) then degrades polyunsaturated fatty acids by catalyzing double bonds at the even-numbered position, followed by isomerization by enoyl-CoA hydratases/isomerases (10 candidate genes). Four putative gene encoding 3-hydroxyacyl-CoA dehydrogenases were identified, responsible for the conversion of the hydroxyl derivatives into the keto. The last step of the first oxidation cycle is predicted to be catalyzed by the thiolase (8 candidate genes).

Mutants with a disruption of the α-methylacyl-CoA racemase (Mcr) gene lost the ability to metabolize poly(*cis*-1,4-isoprene) and related methyl-branched isoprenoid compounds (Arenskötter et al., 2008). Two putative *Mcr* genes were also identified in the genome of *Streptomyces* sp. AC04842.

We found a putative gene encoding an MpaB family protein with 91.83% identity oxygenase mpaB family protein of *Streptomyces* sp. GESEQ-13 (WP_210638107) downstream of *lcp1* on contig 20. This entry represents the catalytic domain found in the endoplasmic reticulum (ER)-bound oxygenases mpaB (MPAB) in the rubber oxygenase (Lcp) from *Streptomyces* sp. K30 (AAR2584), which contains highly conserved arginine and histidine Arg164, Thr168, and His198 residues that are crucial active site residues (Ilcu et al., 2017).

SodA is believed to serve as a radical scavenger during the degradation of poly(*cis*-1,4-isoprene), as the formation of SodA is induced during growth on rubber (Schulte et al., 2008). One putative *SodA* gene was identified.

Presence of these genes (Supplementary Table 2) in the genome of *Streptomyces* sp. AC04842 suggests that they may share the same rubber degradation pathway as predicted previously.

### Transcriptional Induction of Putative Latex Clearing Protein Genes of *Streptomyces* sp. AC04842 When Cultivated With Natural Rubber and Vulcanized Rubber as the Sole Carbon Source

*Streptomyces* sp. AC04842 draft genome revealed the presence of 2 *lcp* gene homologs. To determine whether the transcription of *Streptomyces* sp. AC04842 *lcp1* and *lcp2* putative genes was induced in response to NR and VR. Total RNA was harvested from cells grown on MSM with different polyisoprenes. Total RNA was later converted into cDNA for qPCR studies. Primers used in qPCR validation analysis were verified by checking the primers’ amplification efficiency and specificity. Housekeeping genes using 16S rRNA and the mRNA expression levels were calculated as a ratio of 16S rRNA gene expression. The Ct value for housekeeping genes was consistent among different rubber samples, with a minimal variation of ± 2.

**FIGURE 4:**
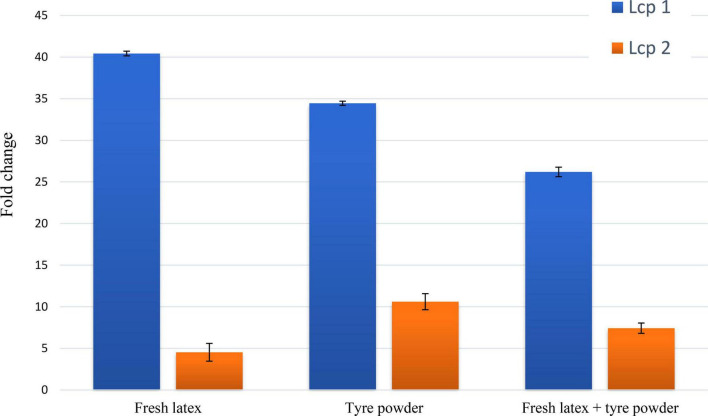
Quantification of expression levels for *Streptomyces* sp. AC04842 *lcp1* (blue bar) and *lcp2* (orange bar) putative genes. Total RNA was isolated from *Streptomyces* sp. AC04842 cultivated with mineral salts medium (MSM) containing fresh latex, tire powder, and both fresh latex with tire powder, as the sole carbon source. The data are mean values ± standard deviations for three independent experiments. The mRNA expression levels were calculated based on the fold change formula of comparative Ct values. Expression values of putative *lcp* genes were compared with an endogenous control (*Streptomyces* sp. AC04842 cultivated in glucose as the sole carbon source).

Cells grown in MSM with glucose (0.2% v/v) without any polyisoprene showed low transcription levels of *lcp1* and *lcp2* genes. In contrast, the transcription levels of *lcp1* and *lcp2* genes in cells grown with polyisoprene were 4–40-fold higher (Figure 4). In general, *lcp1* gene was highly expressed under all 3 conditions compared with *lcp*2. Lcp1 seems to be the key enzyme in rubber degradation. The data are mean values ± standard deviations for three independent experiments with 3 replicates.

### Utilization of Rubber by *Streptomyces* sp. AC04842

For biodegradation studies, *Streptomyces* sp. AC04842 (1 × 10^6^ spores ml^–1^) were transferred into ISP2 broth and incubated at 28°C and 180 rpm until cultures were actively growing by day 3. Cultures using fresh latex and latex gloves as the carbon source showed pigmentation in the MSM broth after 60 days, indicating good growth of the strain and production of secondary metabolites (Supplementary Figure 3).

**FIGURE 5:**
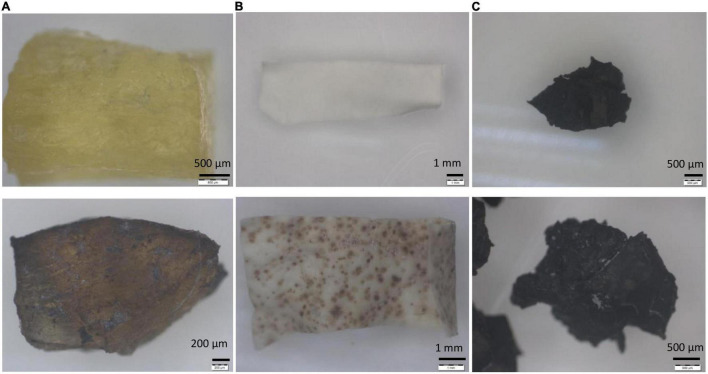
Rubber utilization studies for *Streptomyces* sp. AC04842 cultivate in MSM broth for 60 days at 28°C and 180 rpm, before (top row) and after 60 days (bottom rows). **(A)** Fresh latex pieces, **(B)** latex glove strips, and **(C)** tire granules. Samples were removed from the media, rinsed with 96% ethanol, followed by sterile water, and then left to air-dry. Images with 6.7–40 × magnification were taken using a stereo light microscope (Olympus SZ40).

The steps of biodegradation of any polymer usually start with the microbial attachment on the surface (yellow arrows). Through microscopic and SEM images, the ability of *Streptomyces* sp. AC04842 to attach onto the rubber materials was visible as dark gray deposits (Figure 5).

**FIGURE 6:**
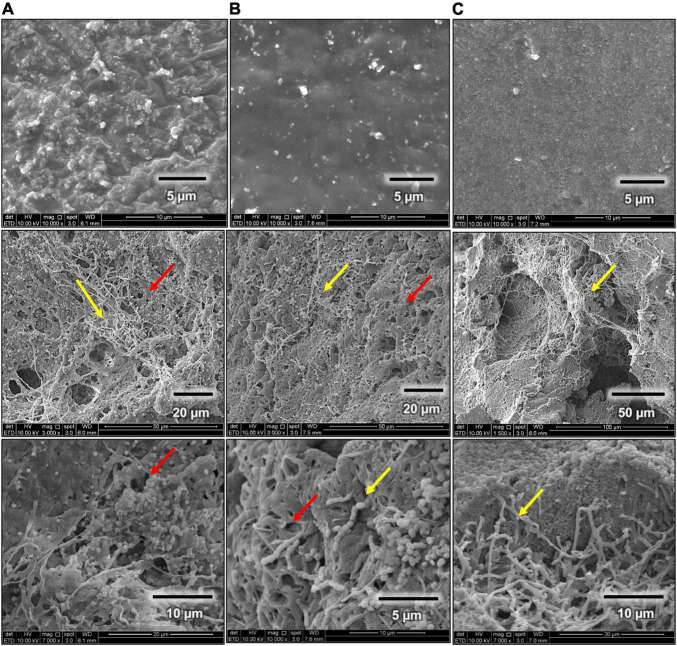
Scanning electron micrographs (SEM Quanta FEG 650) of rubber utilization studies in MSM broth for 60 days at 28°C and 180 rpm, before (first row) and after 60 days (second and third rows). **(A)** Fresh latex pieces, **(B)** latex glove strips, and **(C)** tire granules.

Scanning electron microscope images for rubber materials inoculated with *Streptomyces* sp. AC04842 after 60 days showed that the strain was able to grow and utilize fresh latex, latex glove, or tire as the sole carbon source when compared with control samples (Figure 6). Among the 3 samples, fresh latex showed the most colonization through the presence of mycelia (yellow arrow) compared with latex gloves. The tire sample showed the least colonization. *Streptomyces* sp. AC04842 was found growing in-between gaps of the tire sample (Figure 6). Gaps on the tire sample surface allow better attachment of *Streptomyces* mycelia. Once attached, the microorganism releases degrading enzymes through its mycelia, initiating the first step of rubber degradation (Figure 6). Excretion of enzymes creates holes in the polymer due to the effect of degradation (red arrow) (Figure 6). Long polymer chains are degraded into shorter chains, dimers, and monomers, which are then absorbed into cells and are utilized as carbon and energy sources, producing CO_2_, H_2_O, and CH_4_.

### Attenuated Total Reflection-Fourier Transform Infrared of Rubber Samples Incubated With *Streptomyces* sp. AC04842

Fourier transform infrared spectroscopy is a useful tool to determine the formation or disappearance of functional groups of materials used in the identification of biodegradability. Lcp catalyzes the oxidative C–C cleavage of poly(*cis*-1,4-isoprene) in synthetic rubber and in natural rubber by the addition of oxygen (O_2_) to the double bonds, leading to a mixture of oligonucleotide-isoprenoids with terminal keto and aldehyde groups (endo-type cleavage) (Birke and Jendrossek, 2014). The cleavage products are of different lengths, ranging from C_20_ (four isoprene units) to higher oligo-isoprenoids (Röther et al., 2017).

**FIGURE 7:**
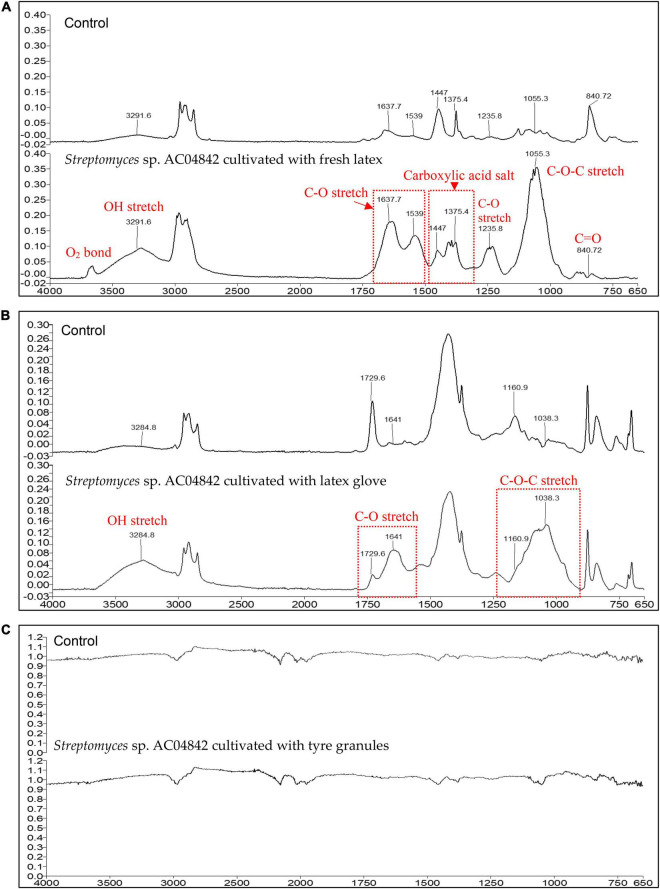
ATR-FTIR profile for **(A)** fresh latex pieces, 5 mg; **(B)** latex glove strips, 1 mg; and **(C)** tire granules, 2 mg, as carbon source in MSM broth for 60 days at 28°C and 180 rpm. ATR-FTIR Spectrum 400 (Perkin Elmer), equipped with ATR ranging from 4,000 to 650/cm (4/cm resolution). Changes in peak profile compared with control are recorded as changes in the presence of functional groups (red fonts) as an indication of rubber polymer biodegradation.

Fresh latex showed the most changes in the IR spectra compared with control (Figure 7A). Characteristic bands of the polyisoprene chain at 2,900–2,800/cm were present, and degradation of fresh latex resulted in the appearance of hydroxyl, carbonyl (aldehyde, ketone, and/or carboxylic acid), and ester groups (Fainleib et al., 2013). There is a peak presence at about ∼3,600/cm, indicating the existence of oxygen-related bonding. The broadening of IR spectra at ∼3,292/cm indicates the OH stretching. Range between 1,750 and 1,700/cm describes simple carbonyl compounds (ketones, aldehydes, esters, or carboxyl). Peak below 1,700/cm corresponds to carbonyl with amides or carboxylates functional group, while intensity between 1,638 and 1,539/cm is due to C–O stretching. The peak at 1,447 and 1,375/cm is caused by the presence of carboxylic acid salt. The presence of a peak at 1,236/cm is caused by C–O stretching. Strong peak profile at 1,055/cm is caused by C–O–C stretching vibration in esters. Similar changes of profile were also reported in natural rubber after 1 year of aging at temperate temperature compared with control (Craciun et al., 2018). Reduction of peak at 841/cm indicates the reduction of isoprene units, OH, C=O in the rubber material (Fainleib et al., 2013).

Changes in latex glove IR spectra compared with control are seen in Figure 7B. Characteristic bands of the polyisoprene chain at 2,900–2.800/cm were present, and the degradation of latex glove resulted in the appearance of hydroxyl, carbonyl (aldehyde, ketone, and/or carboxylic acid), and ester groups (Fainleib et al., 2013). The broadening of IR spectra at ∼3,285/cm indicates the OH stretching. The range between 1,750 and 1,700/cm describes simple carbonyl compounds (ketones, aldehydes, esters, or carboxyl). The peak below 1,700/cm corresponds to carbonyl with amides or carboxylates functional group. The broadening at 1,730/cm and increased intensity at 1,641/cm is due to C–O stretching. Broadening and increase of peak at 1,161–1,038/cm are caused by C–O–C stretching vibration in esters.

No visible changes were observed for tire samples between control and inoculated samples. In the micrograph images, *Streptomyces* sp. AC04842 was able to colonize tire samples with the presence of abundant mycelial growth (Figure 6). However, this strain required a longer duration to degrade the samples as seen in the IR spectra (Figure 7C).

## Discussion

**FIGURE 8:**
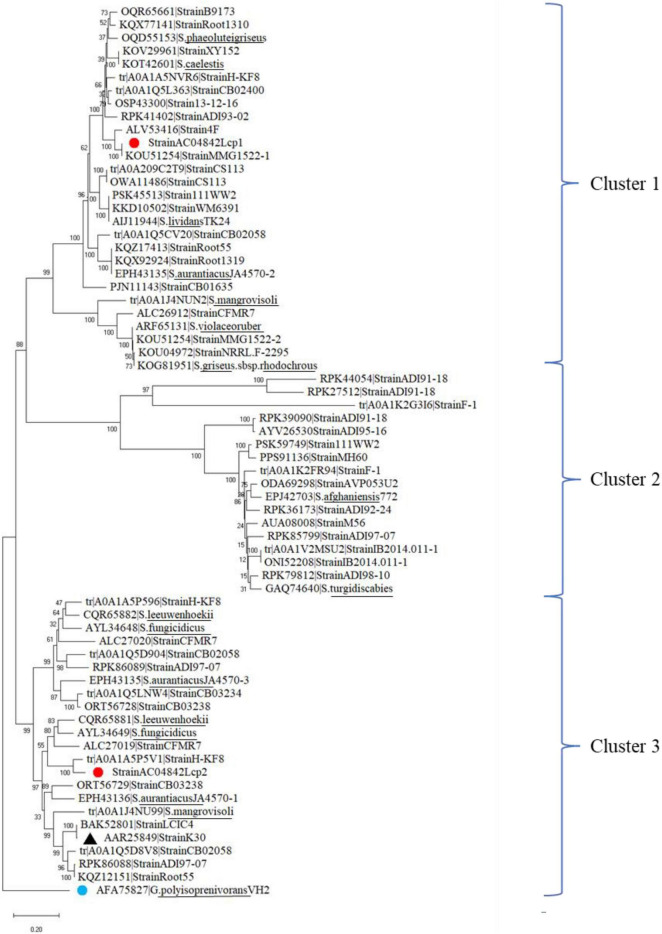
Phylogenetic distribution of 65 lcp amino acids from 45 *Streptomyces* strains obtained from the GenBank, UniProt, and *Streptomyces* sp. AC04842 (red dot). The lcp1 amino acid from *Gordonia polyisoprenivorans* was used as an outgroup (blue dot). The tree was constructed using MEGA X. The bootstrap value was 1,000. The bar below the phylogenetic trees represents the scale of sequence divergence.

From GenBank and UniProt databases, 64 lcp amino acid sequences from 45 *Streptomyces* strains were identified. All the lcp amino acid sequences contained the 13-residue-long highly conserved region. Distribution of lcp amino acid sequences from these *Streptomyces* strains and *Streptomyces* sp. AC04842 (red dot) was analyzed (Figure 8). The lcp sequences were grouped into 3 main clusters. Both *lcp1* and *lcp2* sequences for *Streptomyces* sp. AC04842 were in different clusters (Cluster 1 and 3). Lcp sequence of *Streptomyces* sp. K30 (black triangle) was also clustered under Cluster 3 (Figure 8).

Based on the phylogenetic distribution, *Streptomyces* sp. AC04842 lcp1 sequence is closely related to *Streptomyces* sp. MMG1522. *Streptomyces* sp. MMG1522 was isolated from a soil sample collected in Urbana, IL, United States. Closely related also is an lcp from *Streptomyces* sp. 4F, a strain isolated from the Great Salt Plains of Oklahoma, United States. *Streptomyces* sp. 4F genome contained putative genes encoding Lcp and Mce genes. However, they did not contain isoquinoline 1-oxidoreductase subunit alpha (OxiA) and isoquinoline 1-oxidoreductase subunit beta (OxiB) putative encoding genes.

The lcp2 sequence of *Streptomyces* sp. AC04842 is a closely related lcp from *Streptomyces* sp. HKF-8, a strain that was isolated from Patagonia. *Streptomyces* sp. HKF-8 strain was categorized as an extremophile. Also closely related under the same cluster was lcp1 sequence from *Streptomyces* sp. CFMR 7, a strain isolated from aged latex collected from a rubber tree at Bukit Jambul, Penang, Malaysia.

Based on *Streptomyces* genus observation alone, we can see the diversity of lcp among the strains and their isolation source. In addition to that, within a strain, the Lcp homologs are also unique.

The lcp1 amino acid sequence from *Streptomyces* sp. AC04842 showed 100.00% blast homology to secreted protein (*S. cellulosae* JCM 4462, GHE61056). Lcp2 amino acid sequence from *Streptomyces* sp. AC04842 showed 99.51% blast homology to secreted protein (*S. cellulosae* JCM 4462, GHE38194). Putative *lcp* genes for *S. cellulosae* JCM 4462 were deposited in the GenBank as secreted proteins. OxiA of *Streptomyces* sp. AC04842 protein blast showed 99.37% identity to OxiA of *S. cellulosae* JCM 4462 (GHE38178). OxiB of *Streptomyces* sp. AC04842 protein blast showed 99.48% identity to *S. cellulosae* JCM 4462 (GHE38187). *S. cellulosae* JCM 4462 was described in 1914 by Krainsky under a cooperative description for *Streptomyces* species. Based on taxonomic studies and comparison of rubber-degrading genes operon, *Streptomyces* sp. AC04842 can be identified as *S. cellulosae*. Although there are 16 types of strains for *S. cellulosae*, there have been no studies or reports for the rubber-degrading activity for this species (Parte, 2018). *S. cellulosae* (species and subspecies) have been reported to produce more than 7 useful compounds. Compounds include gabisones, carba sugars, antitumor compound (hexacyclinic acid), proteases, lipases (detergent compatible), phenolic antioxidant compounds, biocontrol compounds (*Ganoderma* and *Sclerotium rolfsii*), and compounds that induce plant resistance against tobacco mosaic virus (Ogawara, 1993; Regina et al., 2000; Muro et al., 2014; Rani et al., 2018; Attia Abo-Zaid et al., 2020; Colobbio et al., 2021; Zulfa et al., 2021).

**FIGURE 9:**
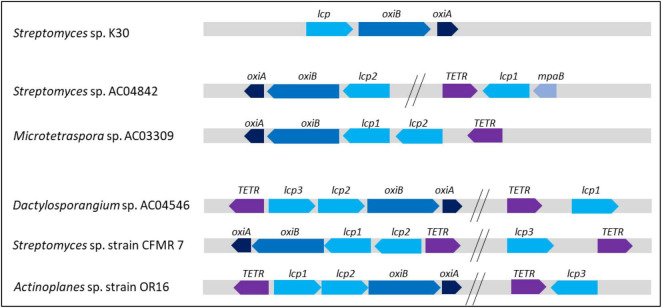
Orientation and position of *Streptomyces* sp. AC04842 and published putative genes encoding Lcp operon. Lcp, OxiB: isoquinoline 1-oxidoreductase beta subunit; OxiA, isoquinoline 1-oxidoreductase alpha subunit; TetR, transcriptional regulator; MpaB, (ER)-bound oxygenases MpaB. Overall comparison of strains with 1 Lcp homolog, *Streptomyces* sp. K30; 2 Lcp homologs, *Streptomyces* sp. AC04842 and *Microtetraspora* sp. AC03309; 3 Lcp homologs, *Dactylosporangium* sp. AC04546, *Streptomyces* sp. strain CFMR 7, and *Actinoplanes* sp. strain OR16.

*Streptomyces* sp. AC04842 Lcp operon is compared with the published operon of *Streptomyces* sp. K30 (Yikmis et al., 2008), *Streptomyces* sp. strain CFMR 7 (Nanthini et al., 2017), and *Actinoplanes* sp. strain OR16 (Gibu et al., 2020; Figure 9). We also included Lcp operon for *Microtetraspora* sp. AC03309 and *Dactylosporangium* AC04546 which we have published recently (Figure 9).

*Streptomyces* sp. K30 has 1 Lcp, while *Streptomyces* sp. strain CFMR 7 and *Actinoplanes* sp. strain OR16 have 3 *lcp* putative genes. In *Streptomyces* sp. K30, *oxiA* gene was located upstream of *oxiB*, which was also located upstream of *lcp* gene. In *Streptomyces* sp. strain CFMR 7 and *Actinoplanes* sp. OR16 which has 3 *lcp* genes, 2 *lcp* genes were located adjacent to each other, followed by *oxiB* and *oxiA* genes. The *lcp3* gene of both strains was located apart from *lcp1* and *lcp2* genes. No *oxiB* and *oxiA* genes were located upstream of the lcp3 *gene*. This is a similar observation seen for the *lcp1* gene in *Streptomyces* sp. AC04842. Despite the absence of *oxiB* and *oxiA* genes near the *lcp1* gene, the TetR-encoding gene was seen directly upstream of the *lcp1* gene for *Streptomyces* sp. AC04842 and *Actinoplanes* sp. strain OR16. The TetR gene was located further away from the lcp3 gene in *Streptomyces* sp. CFMR 7. *Streptomyces* sp. CFMR 7 has a putative gene encoding MpaB with 65.70% similarity to the putative gene encoding MpaB *Streptomyces* sp. AC04842. Location of *mpaB* gene in *Streptomyces* sp. K30 is unknown due to the unavailability of its genome and published information.

The lcp amino acid sequences for *Streptomyces* sp. AC04842, *Microtetraspora* sp. AC03309, and *Dactylosporangium* sp. AC04546 and 248 Lcp amino acid sequences obtained from the GenBank were included for the phylogenetic analysis. Lcp for *Streptomyces* sp. AC04842 (highlighted in gray), *Microtetraspora* sp. AC03309 (highlighted in green), and *Dactylosporangium* sp. AC04546 (highlighted in yellow) was located in different clusters (Supplementary Figure 5). *Microtetraspora* sp. AC03309 lcp1 and *Dactylosporangium* sp. AC04546 Lcp3 seem to be closely related. Pairwise identity for both Lcp was 81.95% similar. *Streptomyces* sp. AC04842 lcp1 and lcp2 amino acid sequences did not cluster together but clustered with lcp from other *Streptomyces* strains.

## Conclusion

*Streptomyces* sp. strain AC04842 was isolated from a soil sample collected in a National Park in Sarawak, East Malaysia. The taxonomic and genomic study suggests that it is similar to *S. cellulosae* JCM 4462. Both strains were very similar, having 2 *lcp* homologs that were located on the separate contigs *oxiA* and *oxiB* genes that were located next to an *lcp* gene. Predicted rubber-degrading genes including crucial active site residues for Lcp (*mpaB*) and radical scavenger gene (*SodA*) were detected. In this study, we reported first the ability of *Streptomyces* sp. AC04842 in utilizing rubber as the sole carbon and energy source. Although lcp1 and lcp2 amino acid sequences shared 77% similarity, functional gene expression revealed that *lcp1* gene is the key enzyme in degrading rubber. Both *lcp* gene expressions were upregulated when cultivated with different rubber sources, namely, fresh latex, latex glove, and tire pieces. The *lcp*2 gene expression increased when cultivated with tire powder, while *lcp1* gene expression was reduced, compared to cultivation with fresh latex. The presence of *lcp* genes in Actinobacteria rubber degraders ranged from 1 to 3 homologs. It would be interesting to explore the functionality of these Lcp homologs in rubber degraders.

## Data Availability Statement

The datasets presented in this study can be found in online repositories. The names of the repository/repositories and accession number(s) can be found at: NCBI—PRJNA665593, MT664881, MT664882, MZ615456, and MZ711223.

## Author Contributions

AB: data curation, writing (original draft), investigation, formal analysis, visualization, and funding acquisition. CT: resources and editing. TY: project administration and funding acquisition. KS: supervision project, conceptualization, and writing (review and editing). All authors have read and agreed to the published version of the manuscript.

## Conflict of Interest

The authors declare that the research was conducted in the absence of any commercial or financial relationships that could be construed as a potential conflict of interest.

## Publisher’s Note

All claims expressed in this article are solely those of the authors and do not necessarily represent those of their affiliated organizations, or those of the publisher, the editors and the reviewers. Any product that may be evaluated in this article, or claim that may be made by its manufacturer, is not guaranteed or endorsed by the publisher.

## Footnotes

1
http://gcycle.com.my/gallery.html

2
www.snapgene.com

3
http://www.bioinformatics.nl/cgi-bin/primer3plus/primer3plus.cgi
