# Supplementary material for: Streptomyces sp. AC04842: Genomic Insights and Functional Expression of Its Latex Clearing Protein Genes (lcp1 and lcp2) When Cultivated With Natural and Vulcanized Rubber as the Sole Carbon Source
Source: Front Microbiol. 2022 May 2;13:854427. doi: 10.3389/fmicb.2022.854427 (PMC9108482; doi:10.3389/fmicb.2022.854427)
Supplement: Supplementary file 1 [file Table_1.pdf]

Appendix M

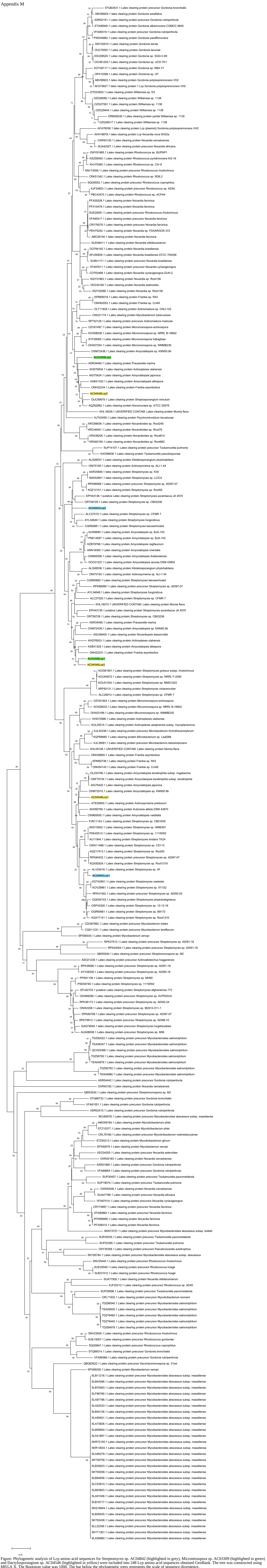

Figure: Phylogenetic analysis of Lcp amino acid sequences for Streptomyces sp. AC04842 (highlighted in grey), Microtetraspora sp. AC03309 (highlighted in green) and Dactylosporangium sp. AC04546 (highlighted in yellow) were included into 248 Lcp amino acid sequences obtained GenBank. The tree was constructed using MEGA X. The Bootstrap value was 1000. The bar below the phylogenetic trees represents the scale of sequence divergence.
