# Supplementary material for: Streptomyces sp. AC04842: Genomic Insights and Functional Expression of Its Latex Clearing Protein Genes (lcp1 and lcp2) When Cultivated With Natural and Vulcanized Rubber as the Sole Carbon Source
Source: Front Microbiol. 2022 May 2;13:854427. doi: 10.3389/fmicb.2022.854427 (PMC9108482; doi:10.3389/fmicb.2022.854427)
Supplement: Supplementary file 2 [file Data_Sheet_1.docx]

**Supplementary Figure 1**. This is an overview of *Streptomyces* sp. AC04842 genomic potential based on RAST server analysis. Subsystem distribution in *Streptomyces* sp. AC04842. Subsystem coverage represented by the green bar indicates the percentage of the proteins included in the subsystems while the subsystem coverage represented by the blue bar refers to the percentage of the proteins that are not included in the subsystems.


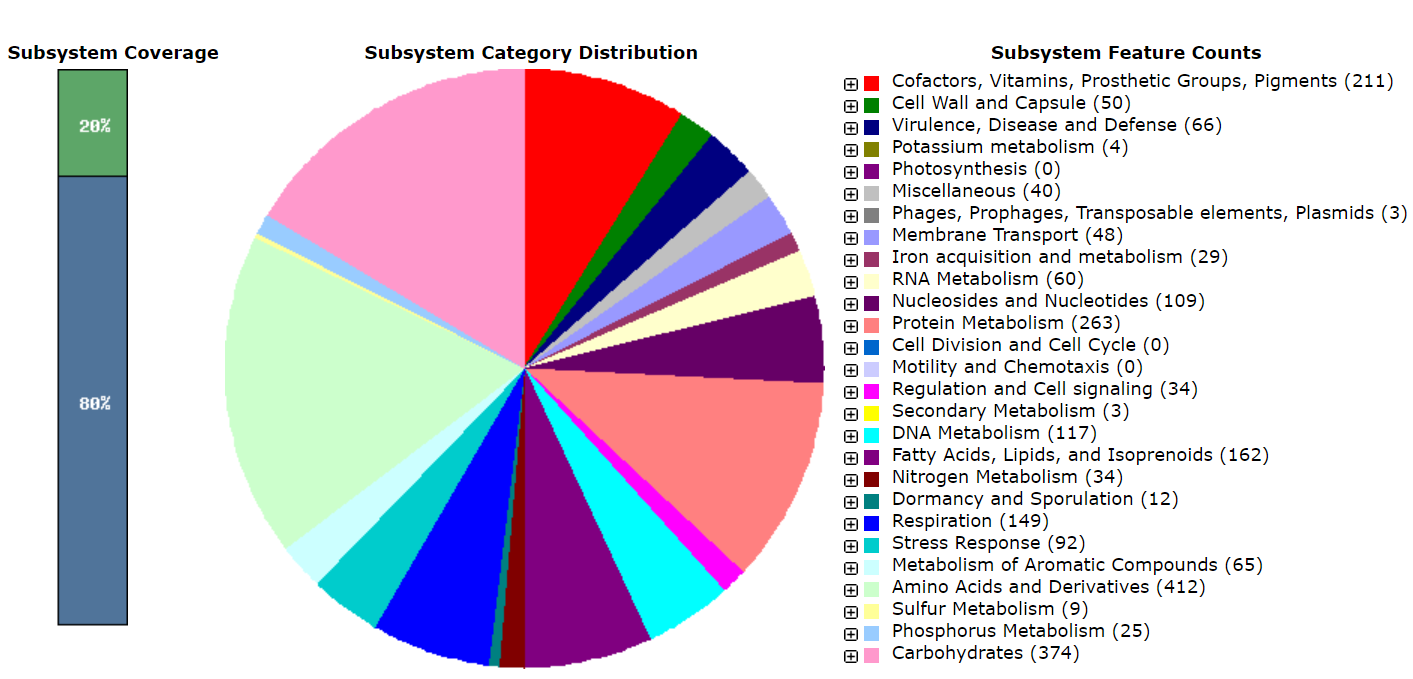


**Supplementary Figure 2.** Cluster of Orthologous (COGs) for *Streptomyces* sp. AC04842 analyzed using egg-NOG mapper v.2 [17]. CELLULAR PROCESSES AND SIGNALING [D] Cell cycle control, cell division, chromosome partitioning, [M] Cell wall/membrane/envelope biogenesis, [N] Cell motility; [O] Post-translational modification, protein turnover, and chaperones, [T] Signal transduction mechanisms, [U] Intracellular trafficking, secretion, and vesicular transport, [V] Defense mechanisms; INFORMATION STORAGE AND PROCESSING [A] RNA processing and modification, [B] Chromatin structure and dynamics, [J] Translation, ribosomal structure and biogenesis, [K] Transcription, [L] Replication, recombination and repair; METABOLISM [C] Energy production and conversion, [E] Amino acid transport and metabolism, [F] Nucleotide transport and metabolism, [G] Carbohydrate transport and metabolism, [H] Coenzyme transport and metabolism, [I] Lipid transport and metabolism, [P] Inorganic ion transport and metabolism, [Q] Secondary metabolites biosynthesis, transport, and catabolism; POORLY CHARACTERIZED, [S] Function unknown, [MC] Multiple classes.

**Supplementary Figure 3.** Cultivation broth from rubber utilization studies. (**a**) MSM media with *Streptomyces* sp. AC04842; (**b**) control, MSM media with fresh latex pieces; (**c**) MSM media with fresh latex pieces and *Streptomyces* sp. AC04842; (**d**) control, MSM media and latex glove strips; (**e**) MSM media with latex glove strips and *Streptomyces* sp. AC04842, (**f**) control, MSM media with tyre granules and (**g**) MSM media with tyre granules and *Streptomyces* sp. AC04842. Cultures using fresh latex and latex gloves as the carbon source showed pigmentation in the MSM broth after 60 days, indicating good growth of the strain and production of secondary metabolites.

Rubber carbon source None Fresh Latex Latex glove Tyre granule

Cultivated with *Streptomyces* sp. AC04842 Yes No Yes No Yes No Yes


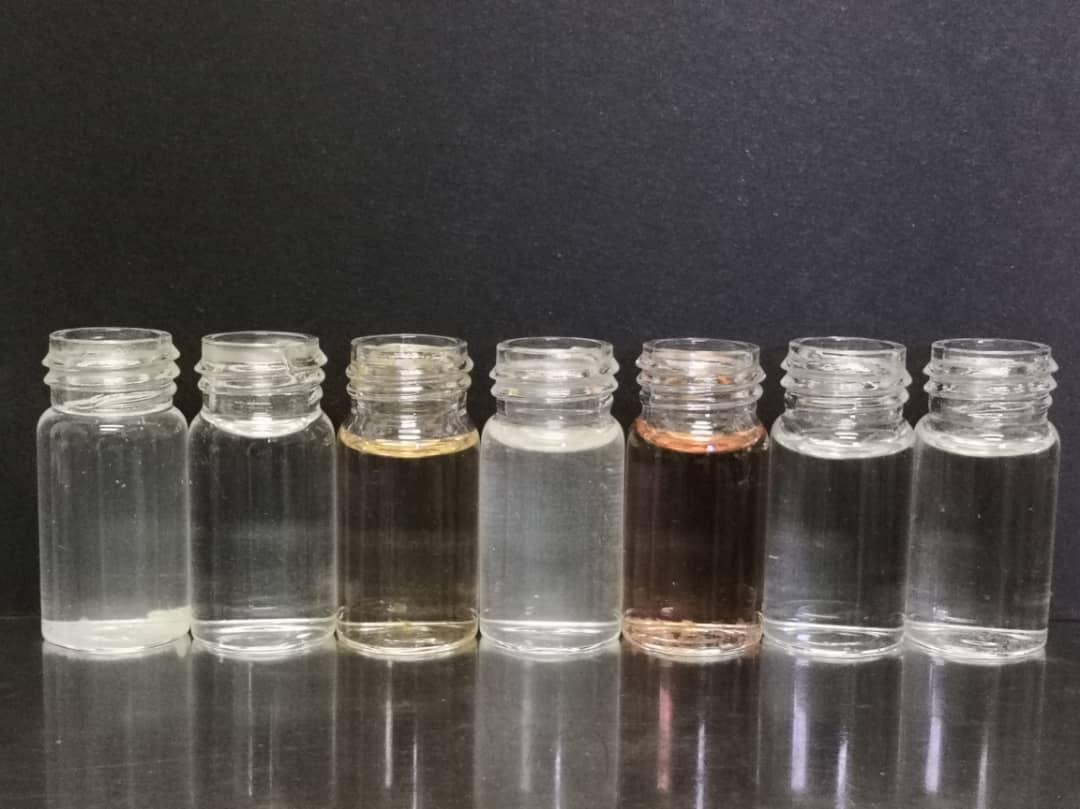


**(a) (b) (c) (d) (e) (f) (g)**

**Supplementary Figure 4.** Location of *lcp1* and *lcp2,* and adjacent genes in *Streptomyces* sp. AC04842 located on chromosome. (A) contig 42: 1, Lanthionine biosynthesis; 2, Lanthionine biosynthesis; 3, D-alanyl-D-alanine carboxypeptidase (EC 3.4.26.4); 5, histone acetyltransferase ; 6, Long chain fatty acid CoA ligase (EC 6.2.1.3); 7, latex clearing protein gene 2 (*lcp2*); 8, Isoquinoline 1-oxidoreductase beta subunit (*oxiB*); 9, Isoquinoline 1-oxidoreductase alpha subunit (*oxiA*); 10, Transcriptional regulator, TETR family; 11, Lipase 2; 12, Hypothetical protein; 13, LD-carboxypeptidase; 14, Protein kinase family protein; 15, Hypothetical protein. (B) Contig 20: 1, secreted alpha amylase; 2, nitroreductase; 3, probably short chain dehydrogenase; 4: hypothetical protein; 5, oxygenase MpaB family protein; 6, latex clearing protein gene 1 (*lcp1*); 7, Transcriptional regulator, TETR family; 8, alcohol dehydrogenase (EC 1.1.1.1); 9, DUF4334 domain containing protein;10, Transcriptional regulator, TETR family; 11, Alpha-methylacyl-CoA-racemase (EC 5.1.99.4); 12, N5,N10-methylenetetraphydromethnopterin reductase related protein, MSMEG 1563.


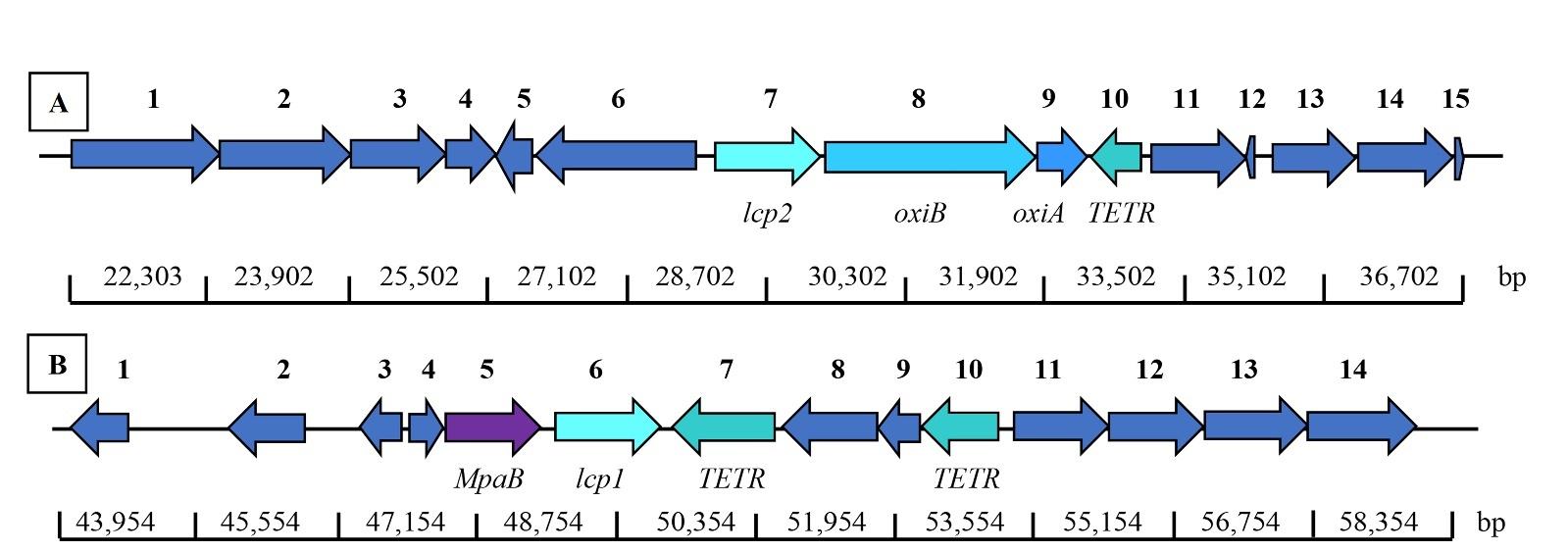


**Supplementary Table 1a to 1d.** Putative speciality genes in the draft genome of *Streptomyces sp.* AC04842.

**Table 1a.** *Streptomyces* sp. AC04842 putative genes related to the chloroaromatic degradation pathway genes (*catA*, *catF*, *catI*, *catJ*, *PCAH*), p-Hydroxybenzoate degradation (*pobA*, *HT*)

|  | Contig | Start (bp) | Stop (bp) | Length (bp) | Putative genes |
| --- | --- | --- | --- | --- | --- |
| 1 | contig000003 | 77,642 | 78,862 | 1221 | 3-ketoacyl-CoA thiolase (EC 2.3.1.16) @ Acetyl-CoA acetyltransferase (EC 2.3.1.9) |
| 2 | contig000004 | 71,437 | 70,220 | 1218 | 3-ketoacyl-CoA thiolase (EC 2.3.1.16) |
| 3 | contig000015 | 21,221 | 19,935 | 1287 | 3-ketoacyl-CoA thiolase (EC 2.3.1.16) @ Acetyl-CoA acetyltransferase (EC 2.3.1.9) |
| 4 | contig000016 | 37,070 | 38,284 | 1215 | 3-ketoacyl-CoA thiolase (EC 2.3.1.16) @ Acetyl-CoA acetyltransferase (EC 2.3.1.9) |
| 5 | contig000020 | 57,171 | 58,385 | 1215 | 3-ketoacyl-CoA thiolase (EC 2.3.1.16) @ Acetyl-CoA acetyltransferase (EC 2.3.1.9) |
| 6 | contig000041 | 3629 | 4429 | 801 | Beta-ketoadipate enol-lactone hydrolase (EC 3.1.1.24) |
| 7 | contig000041 | 10,096 | 8900 | 1197 | O-succinylbenzoate synthase (EC 4.2.1.113) |
| 8 | contig000041 | 42,263 | 41,529 | 735 | Pca regulon regulatory protein PcaR |
| 9 | contig000066 | 13,667 | 14,836 | 1170 | 3-ketoacyl-CoA thiolase (EC 2.3.1.16) @ Acetyl-CoA acetyltransferase (EC 2.3.1.9) |
| 10 | contig000073 | 3574 | 4356 | 783 | Succinyl-CoA:3-ketoacid-coenzyme A transferase subunit A (EC 2.8.3.5) |
| 11 | contig000073 | 4356 | 5024 | 669 | Succinyl-CoA:3-ketoacid-coenzyme A transferase subunit B (EC 2.8.3.5) |
| 12 | contig000073 | 6249 | 7022 | 774 | Protocatechuate 3,4-dioxygenase beta chain (EC 1.13.11.3) |
| 13 | contig000073 | 7029 | 7634 | 606 | Protocatechuate 3,4-dioxygenase alpha chain (EC 1.13.11.3) |
| 14 | contig000073 | 7631 | 8962 | 1332 | 3-carboxy-cis,cis-muconate cycloisomerase (EC 5.5.1.2) |
| 15 | contig000073 | 8959 | 10,080 | 1122 | 4-carboxymuconolactone decarboxylase (EC 4.1.1.44) |
| 16 | contig000075 | 24,517 | 23,294 | 1224 | 3-ketoacyl-CoA thiolase (EC 2.3.1.16) @ Acetyl-CoA acetyltransferase (EC 2.3.1.9) |
| 17 | contig000075 | 24,665 | 25,105 | 441 | Methylmalonyl-CoA epimerase (EC 5.1.99.1); Ethylmalonyl-CoA epimerase |
| 18 | contig000104 | 3595 | 2819 | 777 | 3-oxoadipate enol-lactone hydrolase |
| 19 | contig000117 | 1337 | 39 | 1299 | Beta-ketoadipate enol-lactone hydrolase (EC 3.1.1.24) |
| 20 | contig000120 | 3204 | 4397 | 1194 | 3-ketoacyl-CoA thiolase (EC 2.3.1.16) @ Acetyl-CoA acetyltransferase (EC 2.3.1.9) |
| 21 | contig000120 | 4394 | 5086 | 693 | 3-oxoadipate CoA-transferase subunit A (EC 2.8.3.6) |
| 22 | contig000120 | 5086 | 5748 | 663 | 3-oxoadipate CoA-transferase subunit B (EC 2.8.3.6) |
| 23 | contig000134 | 10,131 | 9061 | 1071 | Muconate cycloisomerase (EC 5.5.1.1) |
| 24 | contig000197 | 5047 | 5919 | 873 | Beta-ketoadipate enol-lactone hydrolase (EC 3.1.1.24) |
| 25 | contig000210 | 3066 | 2854 | 213 | Muconolactone isomerase (EC 5.3.3.4) |
| 26 | contig000223 | 9982 | 8393 | 1590 | Acetyl-coenzyme A carboxyl transferase alpha chain (EC 6.4.1.2) / Acetyl-coenzyme A carboxyl transferase beta chain (EC 6.4.1.2); Propionyl-CoA carboxylase beta chain (EC 6.4.1.3) |
| 27 | contig000391 | 114 | 1499 | 1386 | Acetyl-coenzyme A carboxyl transferase alpha chain (EC 6.4.1.2) / Acetyl-coenzyme A carboxyl transferase beta chain (EC 6.4.1.2); Propionyl-CoA carboxylase beta chain (EC 6.4.1.3) |
| 28 | contig000464 | 746 | 234 | 513 | Uncharacterized ABC transporter, auxiliary component YrbC |
| 29 | contig000003 | 83,029 | 81,659 | 1368 | P-hydroxybenzoate hydroxylase (EC 1.14.13.2) |
| 30 | contig000066 | 27,675 | 26,512 | 1164 | P-hydroxybenzoate hydroxylase (EC 1.14.13.2) |

**Table 1b**. *Streptomyces* sp. AC04842 CRISPR array detected using PATRIC server.

|  | Contig | Start (bp) | Stop (bp) |
| --- | --- | --- | --- |
| 1 | Contig00050 | 24,317 | 25,093 |
| 2 | Contig00083 | 3437 | 4458 |
| 3 | Contig00083 | 15,273 | 15,533 |

**Table 1c**. *Streptomyces* sp. AC04842 antibiotic resistant genes identified from CARD database.

|  | Contig | Start (bp) | Stop (bp) | Amino acid  sequence length (bp) | Drug Class | Identity (%) |
| --- | --- | --- | --- | --- | --- | --- |
| 1 | Contig000021 | 50,283 | 50,654 | 327 | Aminoglycoside antibiotic (ARO:3003395) | 91 |
| 2 | Contig000021 | 53,463 | 54,646 | 1194 | Eflamycin antibiotic (ARO:3003359) | 91 |
| 3 | Contig000054 | 19,487 | 20,545 | 1054 | Macrolide antibiotic (ARO:3003748) | 87 |
| 4 | Contig000056 | 31,394 | 30,192 | 1203 | Macrolide antibiotic (ARO:3000463) | 85 |
| 5 | Contig000539 | 540 | 34 | 507 | Eflamycin antibiotic (ARO:3003368) | 80 |
| 6 | Contig000002 | 36,764 | 34,905 | 1806 | Aminocoumarin antibiotic (ARO:3002522) | 84 |

**Table 1d**. *Streptomyces* sp. AC04842 drug target gene identified using PATRIC server, verified using DrugBank server.

|  | Contig | Product | Start (bp) | Stop (bp) | Length (bp) | Identity |
| --- | --- | --- | --- | --- | --- | --- |
| 1 | Contig000086 | Protein RecA (Q59560) | 26,752 | 27,867 | 1116 | 83 |
| 2 | Contig000145 | 4-hydroxyphenylpyruvate dioxygenase (EC 1.13.11.27) | 114,475 | 12,620 | 11,46 | 91 |
| 3 | Contig000207 | 3-dehydroquinate dehydratase II (EC 4.2.1.10) | 4045 | 4518 | 474 | 87 |
| 4 | Contig000088 | Cell division protein FtsZ | 11,162 | 12,358 | 1197 | 81 |
| 5 | Contig000539 | Translation elongation factor Tu | 540 | 34 | 504 | 80 |
| 6 | Contig000002 | Xylose isomerase (EC 5.3.1.5) | 59,266 | 58,100 | 1167 | 94 |
| 7 | Contig000073 | Endo-1,4-beta-xylanase (EC 3.2.1.8) | 11,605 | 10,154 | 1452 | 83 |
| 8 | Contig000223 | Propionyl-CoA carboxylase beta chain, mitochondrial | 9982 | 8393 | 1509 | 97 |
| 9 | Contig 000372 | Beta-glucosidase | 2151 | 712 | 1440 | 90 |
| 10 | Contig 000393 | Uncharacterized protein | 1080 | 1589 | 510 | 88 |

**Supplementary Table 2a to 2j.** Distribution and loci of β-oxidation related putative genes in the draft genome of *Streptomyces* sp. AC04842**.**

**Table 2a.** *Streptomyces* sp. AC04842 putative transcriptional regulator, TetR family genes on the same contig as *lcp* gene.

|  | Contig | Start (bp) | Stop (bp) | Length (bp) | NCBI Accession Number | Reference gene |
| --- | --- | --- | --- | --- | --- | --- |
| 1 | contig000020 | 51,151 | 49,955 | 1197 | GHE61061 | TetR family transcriptional regulator  [*Streptomyces cellulosae*] |
| 2 | contig000020 | 53,607 | 52,897 | 711 | GHE61077 | TetR family transcriptional regulator  [*Streptomyces cellulosae*] |
| 3 | contig000042 | 33,729 | 33,154 | 576 | WP_210637569 | TetR/AcrR family transcriptional regulator  [*Streptomyces* sp. GESEQ-13] |

**Table 2b.** *Streptomyces* sp. AC04842 putative Twin-arginine translocation protein, TAT protein (TatA, TatB, TatC) genes.

|  | Contig | Start (bp) | Stop (bp) | Length (bp) | NCBI Accession Number | Reference gene |
| --- | --- | --- | --- | --- | --- | --- |
| 1 | contig000028 | 1,533 | 1967 | 435 | GHE51300 | Sec-independent protein translocase protein TatB [*Streptomyces cellulosae*] |
| 2 | contig000042 | 39,296 | 39,057 | 240 | WP_033274310 | Twin-arginine translocase TatA/TatE family subunit [*Streptomyces griseorubens*] |
| 3 | contig000171 | 8448 | 8729 | 282 | WP_024885165 | Sec-independent protein translocase subunit TatA [*Streptomyces*] |
| 4 | contig000171 | 8831 | 9691 | 861 | GHE59274 | Sec-independent protein translocase protein TatC [*Streptomyces cellulosae*] |

**Table 2c.** *Streptomyces* sp. AC04842 putative Acyl-CoA synthase (EC 6.2.1.1) genes.

|  | Contig | Start (bp) | Stop (bp) | Length (bp) | NCBI Accession Number | Reference gene |
| --- | --- | --- | --- | --- | --- | --- |
| 1 | contig000160 | 15,975 | 15,172 | 804 | MUT91853 | Acyl-CoA synthetase [*Streptomyces* sp. Z38] |

**Table 2d.** *Streptomyces* sp. AC04842 putative Acyl-CoA dehydrogenase (EC 1.3.8.7) (EC 1.3.99.2) genes.

|  | Contig | Start (bp) | Stop (bp) | Length (bp) | NCBI Accession Number | Reference gene |
| --- | --- | --- | --- | --- | --- | --- |
| 1 | contig000002 | 11,205 | 12,713 | 1689 | MBM4832232 | Acyl-CoA dehydrogenase family protein [*Actinospica acidiphila*] |
| 2 | contig000056 | 12,049 | 13,683 | 1635 | GHE43051 | Acyl-CoA dehydrogenase  [*Streptomyces cellulosae*] |
| 3 | contig000142 | 12,933 | 14,072 | 1140 | NEC48390 | Acyl-CoA/acyl-ACP dehydrogenase [*Actinospica acidiphila*] |
| 4 | contig000162 | 1098 | 2327 | 1230 | GHE65845 | Acyl-CoA dehydrogenase family protein [unclassified *Streptomyces*] |
| 5 | contig000484 | 671 | 471 | 201 | EXS97612 | Acyl-CoA dehydrogenase, C-terminal domain protein [*Acinetobacter baumannii* 45002_10] |

**Table 2e.** *Streptomyces* sp. AC04842 putative 2,4-dienoyl-CoA reductase (EC 1.3.1.34) genes.

|  | Contig | Start (bp) | Stop (bp) | Length (bp) | NCBI Accession Number | Reference gene |
| --- | --- | --- | --- | --- | --- | --- |
| 1 | contig000002 | 6487 | 7566 | 1080 | SCE07444 | 2,4-dienoyl-CoA reductase  [*Streptomyces* sp. di50b] |
| 2 | contig000032 | 27,294 | 28,412 | 1119 | MBP2400965 | 2,4-dienoyl-CoA reductase-like NADH-dependent reductase (Old Yellow Enzyme family) [*Streptomyces syringium*] |
| 3 | contig000078 | 29,910 | 31,037 | 1128 | SCE08340 | 2,4-dienoyl-CoA reductase  [*Streptomyces* sp. di50b] |
| 4 | contig000281 | 2098 | 83 | 2016 | SCD48527 | 2,4-dienoyl-CoA reductase  [*Streptomyces* sp. di188] |

**Table 2f.** *Streptomyces* sp. AC04842 putative Enoyl-CoA hydratases (EC 4.2.1.17) genes.

|  | Contig | Start (bp) | Stop (bp) | Length (bp) | NCBI Accession Number | Reference gene |
| --- | --- | --- | --- | --- | --- | --- |
| 1 | contig000016 | 55,925 | 55,143 | 783 | GHE65520 | Enoyl-CoA hydratase [Streptomyces cellulosae] |
| 2 | contig000020 | 20,010 | 19,246 | 765 | WP_189909493 | crotonase/enoyl-CoA hydratase family protein [*Streptomyces viridodiastaticus*] |
| 3 | contig000024 | 37,058 | 37,849 | 792 | GGQ77955 | Enoyl-CoA hydratase  [*Streptomyces griseorubens*] |
| 4 | contig000039 | 6109 | 6894 | 786 | WP_210635000 | Enoyl-CoA hydratase-related protein [*Streptomyces* sp. GESEQ-13] |
| 5 | contig000044 | 8628 | 9455 | 828 | WP_019523546 | Enoyl-CoA hydratase family protein [unclassified *Streptomyces*] |
| 6 | contig000051 | 17,010 | 16,243 | 768 | GHE68025 | Enoyl-CoA hydratase [*Streptomyces cellulosae*] |
| 7 | contig000073 | 16,378 | 15,767 | 612 | GHE61397 | Enoyl-CoA hydratase [*Streptomyces cellulosae*] |
| 8 | contig000123 | 3873 | 4676 | 804 | GHE51256 | Enoyl-CoA hydratase [*Streptomyces cellulosae*] |
| 9 | contig000146 | 9638 | 8967 | 672 | GHE26833 | Enoyl-CoA hydratase [*Streptomyces cellulosae*] |
| 10 | contig000222 | 2722 | 3525 | 804 | WP_028960728 | Enoyl-CoA hydratase/isomerase family protein [unclassified *Streptomyces*] |

**Table 2g.** *Streptomyces* sp. AC04842 putative 3-hydroxyacyl-CoA dehydrogenases (EC 1.1.1.-) genes.

|  | Contig | Start (bp) | Stop (bp) | Length (bp) | NCBI Accession Number | Reference gene |
| --- | --- | --- | --- | --- | --- | --- |
| 1 | contig000003 | 28,656 | 30,170 | 1515 | GHE31118 | 3-hydroxyacyl-CoA dehydrogenase  [*Streptomyces cellulosae*] |
| 2 | contig000004 | 70,223 | 68,094 | 2130 | GHE43722 | 3-hydroxyacyl-CoA dehydrogenase [*Streptomyces cellulosae*] |
| 3 | contig000016 | 38,319 | 40,499 | 2181 | GHE65596 | 3-hydroxyacyl-CoA dehydrogenase [*Streptomyces cellulosae*] |
| 4 | contig000142 | 17,474 | 16,086 | 1389 | MBT0126160 | 3-hydroxyacyl-CoA dehydrogenase [*Streptomyces* sp. CPA0001] |

**Table 2h.** *Streptomyces* sp. AC04842 putative Thiolase/ 3-ketoacyl-CoA thiolase (EC 2.3.1.16) (EC 2.3.1.9) genes.

|  | Contig | Start (bp) | Stop (bp) | Length (bp) | NCBI Accession Number | Reference gene |
| --- | --- | --- | --- | --- | --- | --- |
| 1 | contig000066 | 13,667 | 14,836 | 1170 | WP_210637043 | Thiolase family protein  [*Streptomyces* sp. GESEQ-13] |
| 2 | contig000120 | 3204 | 4397 | 1194 | WP_210637463 | Thiolase family protein  [*Streptomyces* sp. GESEQ-13] |
| 3 | contig000003 | 77,642 | 78,862 | 1221 | GHE26124 | Acetyl-CoA acetyltransferase  [*Streptomyces cellulosae*] |
| 4 | contig000004 | 71,437 | 70,220 | 1218 | GHE43716 | Acetyl-CoA acetyltransferase  [*Streptomyces cellulosae*] |
| 5 | contig000015 | 21,221 | 19,935 | 1287 | GHE62225 | Acetyl-CoA acetyltransferase  [*Streptomyces cellulosae*] |
| 6 | contig000016 | 37,070 | 38,284 | 1215 | GHE65602 | Acetyl-CoA acetyltransferase  [Streptomyces cellulosae] |
| 7 | contig000020 | 57,171 | 58,385 | 1215 | GHE61105 | Acetyl-CoA acetyltransferase  [*Streptomyces cellulosae*] |
| 8 | contig000075 | 24,517 | 23,294 | 1224 | GHE68890 | Acetyl-CoA acetyltransferase  [*Streptomyces cellulosae*] |

Acetyl-CoA acetyltransferase belongs to Thiolase family.

**Table 2i.** *Streptomyces* sp. AC04842 putative Endoplasmic reticulum (ER)-bound oxygenases mpaB (MPAB) genes.

|  | Contig | Start (bp) | Stop (bp) | Length (bp) | NCBI Accession Number | Reference gene |
| --- | --- | --- | --- | --- | --- | --- |
| 1 | Contig000020 | 47,518 | 48,441 | 924 | WP_215188902 | Oxygenase MpaB family protein  [*Streptomyces* sp. McG8] |

**Table 2j.** *Streptomyces* sp. AC04842 putative SodA, Nickel-dependent superoxide dismutase (EC 1.15.1.1) genes.

|  | Contig | Start (bp) | Stop (bp) | Length (bp) | NCBI Accession Number | Reference gene |
| --- | --- | --- | --- | --- | --- | --- |
| 1 | contig000008 | 60,080 | 59,685 | 396 | WP_006136256 | Superoxide dismutase, Ni [*Streptomyces*] |
